# Isolated hypoaldosteronism as first sign of X-linked adrenal hypoplasia congenita caused by a novel mutation in NR0B1/DAX-1 gene: a case report

**DOI:** 10.1186/s12881-019-0834-7

**Published:** 2019-06-04

**Authors:** Lorenzo Iughetti, Laura Lucaccioni, Patrizia Bruzzi, Silvia Ciancia, Elena Bigi, Simona Filomena Madeo, Barbara Predieri, Florence Roucher-Boulez

**Affiliations:** 1 Pediatric Unit, Department of Medical and Surgical Sciences of the Mother, Children and Adults, University of Modena and Reggio Emilia, Via del Pozzo n. 71, 41124 Modena, Italy; 2 Laboratoire de Biochimie et Biologie Moléculaire Grand Est, UM Pathologies Endocriniennes Rénales Musculaires et Mucoviscidose, Groupement Hospitalier Est, Hospices Civils de Lyon, Univ Lyon, Université Claude Bernard Lyon 1, Lyon, France

**Keywords:** *NR0B1*, *DAX-1*, X-linked adrenal hypoplasia congenita, Adrenal insufficiency, Hypoaldosteronism, Mineralocorticoid deficiency

## Abstract

**Background:**

X-linked Adrenal Hypoplasia Congenita (AHC) is a rare cause of primary adrenal insufficiency due to mutations in the *NR0B1* gene, causing a loss of function of the nuclear receptor protein *DAX-1*. Adrenal insufficiency usually appears in the first 2 months of life, but can sometimes emerge during childhood. Hypogonadotropic Hypogonadism is often associated later in life and patients may develop azoospermia. We describe an unusual onset of AHC started with isolated hypoaldosteronism as first and only sign of the disease.

**Case presentation:**

A 18-days-old newborn presented with failure to thrive and feeding difficulties. Blood tests showed severe hyponatremia, hyperkalemia and hypochloremia. Renin was found over the measurable range and aldosterone was low whereas cortisol level was normal with a slightly increased ACTH. In the suspicion of Primary Hypoaldosteronism, correction of plasmatic electrolytes and replacement therapy with Fludrocortisone were promptly started. The subsequent evidence of low plasmatic and urinary cortisol and increased ACTH required the start of Hydrocortisone replacement therapy and it defined a clinical picture of adrenal insufficiency. Genetic analysis demonstrated a novel mutation in the *DAX-1* gene leading to the diagnosis of AHC.

**Conclusions:**

AHC onset may involve the aldosterone production itself, miming an isolated defect of aldosterone synthesis. *NR0B1/DAX-1* mutations should be considered in male infants presenting with isolated hypoaldosteronism as first sign of adrenal insufficiency.

## Background

X-linked Adrenal Hypoplasia Congenita (AHC; OMIM #300200) is a congenital disorder characterized by adrenal insufficiency, often associated with hypogonadotropic hypogonadism. The first case, described in 1948, was a male who died at 33 days of life for adrenal crisis with salt wasting [1]. The estimated incidence is 1 in 12,500 births. AHC results from mutations in the *NR0B1/DAX-1* gene, a nuclear receptor located on the X-chromosome (Xp21) and expressed in adrenal cortex, gonads, hypothalamus and pituitary gland. *DAX-1* regulates adrenal and reproductive differentiation and function, although its role is not completely clear [2]. *DAX-1 *mutations are usually associated with primary adrenal failure, hypogonadotropic hypogonadism and impaired spermatogenesis (oligospermia or azoospermia are typically present) although uncommon phenotypes, different clinical features and different age of onset have been described [2, 3]. More than 100 pathogenic mutations of *DAX-1* are known, together with deletions of the exons or of the entire gene, most of which are located at the carboxyl-terminal of the protein and are nonsense or frameshift missense mutations. We present a newborn with isolated hypoaldosteronism as first sign of AHC caused by a novel mutation (c.848_849delinsCC) of *DAX-1* gene [4].

## Case presentation

The male 18-days-old Caucasian newborn was admitted to our department for ineffective breastfeeding and failure to thrive. He was born at term from spontaneous delivery, small for gestational age to non-consanguineous parents [5]. Pregnancy was uneventful except for a delayed intrauterine growth restriction detected during the last month of gestation. External genitalia were of normal appearance, prepubertal, with testicular volumes of 1 ml. Blood tests showed a severe hyponatremia (Na ^+^ 110 mEq/l; NR 136–146 mEq/l), hyperkalemia (K^+^ 7.5 mEq/l; NR 3.5–5.30 mEq/l), hypochloremia (Cl^-^ 81 mEq/l; NR 97–110 mEq/l) and metabolic acidosis with increased lactate. Glycemia was within the normal range for age (68 mg/dl); urinary sodium loss was also detected (Natriuria 16 mEq/l).

Endocrinological tests revealed low plasmatic aldosterone levels (38.6 pg/ml; NR 50–300 pg/ml), dramatic increased renin (44,100 μU/ml; NR 4.4–46.1), elevated levels of adrenocorticotropic hormone (ACTH, 91.4 pg/ml NR 4,3–52) and normal plasmatic cortisol (13.7 μg/dl NR 6.7–22.6). Skin pigmentation was normal except for mild pigmentation of the external genitalia. Neonatal screening for 17-OH-progesterone was within the normal range. Electrolytes replacement first intravenous and then oral, and therapy with Fludrocortisone (50 μg/die) and salt integration were started with normalization of clinical and hormonal conditions. Treatment revealed to be effective and the newborn started growing properly, with normal electrolytes. Cerebral ultrasounds and cerebral magnetic resonance (MRI) were normal, as well as the elettrocardiogram (ECG) and chest X-rays. Abdominal MRI showed normal size of the adrenal glands. A diagnosis of Primary Hypoaldosteronism was entertained and genetic analysis of the *CYP11B2* gene (encoding aldosynthase) was requested.

During the follow up after the first 5 months of life, ACTH levels started increasing again although there was a good treatment compliance, normal electrolytes, good weight and length gain and slightly low basal cortisol levels, within the normal range for age (Table 1).

**Table 1:** Medical history timeline where auxological parameters during the first 2 years of life and biochemical and hormonal values are summarized.

Chronologic Age (years)	38 + 2 weeks of GA (Birth)	40 + 2 weeks of GA (hospital admission)	0.3	0.47	0.6	0.8	1.2	1.77	2.1
Length (cm) (°p)^a^	/	/	57.6 (1.6)	62.3 (1.5)	65.9 (5)	70 (10)	76 (16)	82.6 (17)	85.3 (18)
Weight (Kg) (°p)^a^	2.505 (5)	2.450 (<< 3)	4.81 (< 3)	5.66 (< 3)	6.46 (< 3)	7.51 (3–10)	9.47 (47)	11 (57)	12.2 (53)
Head Circumference (cm)	/	/	39	41	42.5	44	46	47	48
Pubertal Stage (testicular volume)	/	P1,G1,A0 (1 ml bilateral)	P1,G1,A0 (1 ml bilateral)	P1,G1,A0 (1 ml bilateral)	P1,G1,A0 (1 ml bilateral)	P1,G1,A0 (1 ml bilateral)	P1,G1,A0 (1 ml bilateral)	P1,G1,A0 (2 ml bilateral)	P1,G1,A0 (2 ml bilateral)
ACTH (pg/ml)	/	91.4	/	124.9	/	300	83.1	40.6	81.6
Cortisol	/	13.7	/	4.4	5.2	7.4	19.4	13.2	19.5
Aldosterone (pg/ml)	/	38.6	/	55.9	45.2	21.5	16.7	16.4	15.2
Renin (μU/ml)	/	44,100	/	181.2	/	123.6	5.6	2.5	23.4
17 OH progesterone (ng/ml)	/	4.4	/	/	/	0.4	/	0.1	0.1
Na (mEq/L)	/	110	138	134	139	139	140	138	139
K (mEq/L)	/	7.5	5.7	4.9	5	4.2	4.8	4.5	4.3
DHEA-S (μg/ml)	/	1.09	/	/	/	0.03	/	< 0.02	< 0.02
Testosterone (ng/ml)	/	/	/	/	/	0.2	/	/	< 0.1
Treatment	/	Fludro-cortisone 50 mcg/day + NaCl 11.6% 1 ml × 7/day	Fludro-cortisone 100 mcg/day + NaCl11.6% 1 ml × 7/day	Fludro-cortisone 100 mcg/day + NaCl11.6% 1 ml × 7/day	Fludro-cortisone 100 mcg/day + NaCl11.6% 1 ml × 7/day	Started Hydro-cortisone 10 mg/m2/day +Fludro-cortisone 100 mcg/day + NaCl11.6% 1 ml × 7/day	Hydro-cortisone 10 mg/m2/day + Fludro-cortisone 100 mcg/day + NaCl11.6% 1 ml × 7/day	Hydro-cortisone 10 mg/m2/day +Fludro-cortisone 100 mcg/day + NaCl11.6% 1 ml × 7/day	Hydro-cortisone 10 mg/m2/day +Fludro-cortisone 100 mcg/day + NaCl11.6% 1 ml × 7/day

^a^Percentiles referred to the WHO growth charts for male infants (https://www.who.int/childgrowth/standards/en/)

At 9 months of age the result of the *CYP11B2* gene sequencing did not show any mutation. The parents refused to accomplish a short synacthen test (SST), however basal ACTH was 300 pg/ml and 24-h urine collection pointed out low levels of urinary cortisol (20 μg/24 h, NR 58–403 μg/24 h) with normal Na/K urinary ratio. Moreover, normal serum 17-OH-progesterone was found, ruling out the hypothesis of congenital adrenal hyperplasia (CAH).

Luteinizing hormone (LH) and follicle-stimulating hormone (FSH) were found, respectively, of 1 IU/l and 2.2 IU/l, and Testosterone of 0.2 ng/ml, being to the highest levels of the normal range for age.

**Fig. 1:**
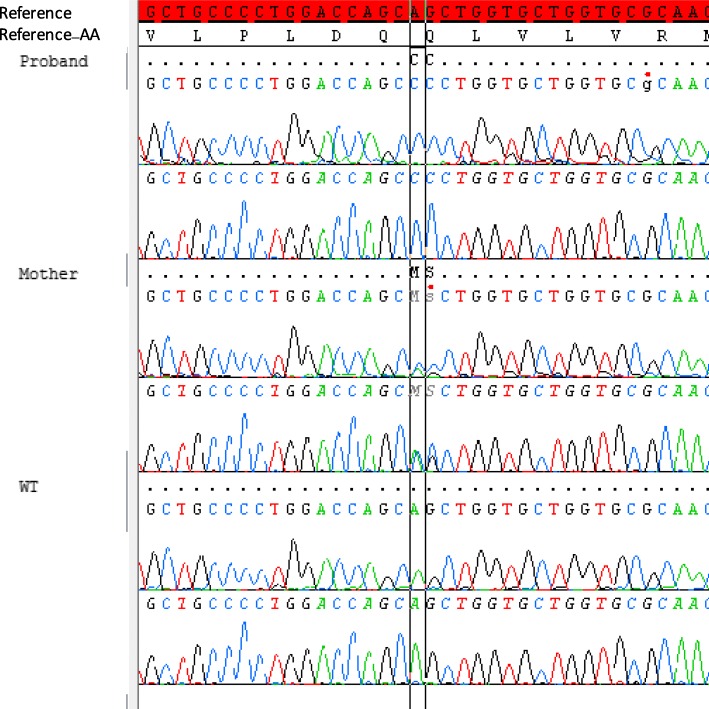
Partial chromatograms showing the novel *NR0B1 /DAX-1* mutation. Base change c.848_849delinsCC leading to the missense mutation p.(Gln283Pro). The reference sequence is in highlighted in red. The proband’s DNA sequence is above, the mother’s DNA sequence in the middle and a healthy control’s sequence underneath

A diagnosis of adrenal insufficiency was made and replacement therapy with Hydrocortisone (10 mg/m2/day), joint to already in use Fludrocortisone therapy (100 μg/day) and salt integration, was started. An abnormal development of adrenal glands rather than an enzymatic defect was hypothesized. Adrenal antibodies and very long chain fatty acids (VLCFA) were negative. DNA analysis performed by Sanger sequencing identified a novel in frame indel mutation in the *NROB1* gene (c.848_849delinsCC or p.(Gln283Pro)), confirming the diagnosis of AHC. As expected the same mutation was carried by the mother as hemizygous (see Fig. 1).

## Discussion

We describe the case of a newborn presenting with unspecific symptoms and silent family history, in which AHC started with an isolated mineralocorticoid deficiency, leading to the initial hypothesis of primary hypoaldosteronism treated with fludrocortisone. The negative results of *CYP11B2* genetic analysis and the tightened endocrinological follow-up allowed to discover the secondary development of glucocorticoid deficiency, although parents refused to perform a SST, and a prompt supplementation therapy with hydrocortisone was started before any adrenal crisis. The initial presentation of pathogenic *DAX-1* mutations is often a combination of mineral and glucocorticoid deficiency but, especially during the neonatal period, it is known how aldosterone deficiency may precede cortisol deficiency at onset, confounding the initial diagnosis. The reason of this peculiar clinical appearance is still unknown, and recent studies failed to establish a genotype phenotype correlation in patients with *NR0B1* mutations [6].

Generally, healthy newborns exhibit a renal tubular immaturity at birth, with sodium wasting and impaired water reabsorption. Renal sodium reabsorption is mainly regulated by aldosterone, through the binding of its receptor, the mineralocorticoid receptor (MR), a transcription factor regulating the expression of several transporting proteins related to sodium homeostasis [7]. Soon after birth, changing from an intra-uterine aquatic environment to the out-womb/terrestrial one, a partial aldosterone resistance is well documented, with high plasma levels of aldosterone and renin, contrasting with biological signs of functional hypoaldosteronism (as hyponatremia, hyperkalemia and urinary sodium loss). The highest aldosterone levels detected in the cord blood at birth are consistent with the de novo synthesis in the fetal adrenal gland, given the very early expression of the aldosterone synthase gene starting from 13 gestational weeks [8]. This hormonal resistance could account for the weight loss observed during the first days of life and might be an adaptive phenomenon of the passage from the in utero to terrestrial life [9]. Nevertheless, the transient aldosterone resistance is associated with low MR renal presence at birth, followed by a significant increase in the postnatal period, with a complete renal tubular expression developed during the first year of life, contemporary to renal functional maturation [10]. It was demonstrated that high aldosterone levels soon after birth are required for the MR optimal up-regulation in the postnatal period, and it is therefore indispensable for sodium homeostasis [7]. Glucocorticoids are able to bind MR with the same affinity of mineralocorticoids, being a possible responsible for the development of MR during the first year of life even in cases of hypoaldosteronism. Moreover, basal cortisol levels at birth and during the first days/months of life may be normal in patients with AHC, sign of the possible adrenal transition from fetal to adult zone. In fact, several adrenal enzymes responsible for steroids synthesis of the fetus, are active since the very first weeks of gestation, and may lead to the development of an “adrenal reserve”, during the neonatal and perinatal period, responsible for the slow development of glucocorticoid deficiency symptoms.

It is important to bear in mind that in boys, once CAH has been ruled out, the most frequent cause of adrenal insufficiency in neonatal period are the *DAX-1* mutations. In our case, CAH was excluded by normal 17-OH-progesterone levels at neonatal screening [11–13]. Afterwards, the DNA sequencing identified a novel in frame indel mutation in the NR0B1 gene and the diagnosis of AHC was made. An indel was identified with the deletion of two base-pair (bp) replaced by two cytosine in position 848_849. The sequence variants is designated according to the Human Genome Society recommendations (https://www.hgvs.org) using the National Center for Biotechnology Information (NCBI) reference sequences NM_000475.4, NP_000466.2 built on the GRCh37/hg19 and is NM_000475.4:c.848_849delinsCC or p.(Gln283Pro).

Pathogenicity prediction was performed in silico using several programs: Align-GVGD, Polyphen-2 and SIFT and the variant was predicted to be most likely pathogenic using Align-GVGD class, probably damaging, using Polyphen-2, deleterious using SIFT. The Grantham score that ranges from 0 to 215, was calculated to predict the effect of substitutions between amino acids based on chemical properties (i.e. polarity and molecular volume). Higher scores indicate greater differences between two amino acids and may indicate a stronger (negative) effect on protein structure and function. Physicochemical effect of this variation is important with a Grantham score of 76. Frequency databases (dbSNP, ESP, and gnomAD) were searched to determine if the variant had already been reported and it was not. It has not been found in 100 Caucasian healthy controls sequences. According to the ACMG/AMP standards Guidelines the variant is classified as pathogenic [14].

Since the first description of the *NR0B1/DAX-1* mutation in 1994 as the etiology of AHC, several novel mutations have been discovered. Deletions, nonsense and frameshift mutations of the carboxyl-terminal domain seem to be the most common and are usually associated to clinical phenotypes. Structure-function studies suggest that mutations in the amino-terminal domain are compensated by the presence of redundant LXXLL motifs that allow *DAX-1* interacting with other proteins, changing the normal nucleocytoplasmic ratio. Stuart et al. described an amino-terminal *DAX-1* missense mutation causative of AHC associated with isolated mineralocorticoid deficiency [15].

The different causative mutations of *DAX-1* can be responsible of the phenotypical variability, but different clinical features between patients of the same family, carrying the same mutations, indicate that environmental factors are involved and a detailed study of this disease also in presence of mild symptoms is essential to make a correct diagnosis and start a prompt therapy [16–18]. Table 2 summarizes some of the cases of *DAX-1* mutation with initial isolated hypoaldosteronism and the main clinical and genetic characteristics described in literature [11–13, 15–24].

**Table 2:** Recent cases of ACH described in literature: main clinical and genetic characteristics

References	Age at presentation	Age at diagnosis	Presentation	Family history	Genetic analysis
Evliyaoglu O, 2013 [11]	33 days		↓Na,↑K, ↓aldosterone, ↑renin, ↑ACTH, ↓cortisol	Both parents normal	c.543delA
Abraham MB, 2016 [12]	5 years	5 years	↓Na,↑K, ↓aldosterone, ↑ACTH, ↓cortisol	Mother as a carrier	c.844C > T
Wheeler B, 2008 [13] case 1	6 weeks	6 weeks	↓Na,↑K, aldosterone N, ↑renin, ↑ACTH, ↓cortisol		Nucleotide transversion c.192C > A, c.498G > A
Wheeler B, 2008 [13] case 2	18 months	24 months (primary adrenal insufficiency), 16.5 years (AHC with DAX1 mutation)	↓Na,K n, aldosterone undectable, ↑renin, ↓cortisol		51 bp deletion nt1068–1118
Wheeler B, 2008 [13] case 3	1 years	4 years (primary adrenal insufficiency), 25 years (AHC with DAX1 mutation)	↓Na,↑K, ↓cortisol response to ACTH test, ↑ACTH		8 bp deletion nt 1181–1188
Verrijn Stuart AA, 2007 [15]	4 weeks	4 weeks misdiagnosed as CAH, 11 years diagnosis of AHC	↓Na,↑K, ACTH n, cortisol n	Mother as a carrier	W105C TGG➔TGC (missense mutation in the amino-terminal region
Calliari LE, 2013 [16]		8 years	Na n,K n, ↓ aldosterone, ↓cortisol	2 younger brothers	Transition C➔Tand stop codon at 359 (Q359X)
Zhang Z, 2015 [17] case 1	9 years	9 years diagnosis of primary adrenal insufficiency, > 23 years diagnosis of AHC	Na n,K n, ↑ ACTH, ↓cortisol	Mother as a carrier, brother affected	c.1268delA
Zhang Z, 2015 [17] case 2	8 years	8 years diagnosis of primary adrenal insufficiency, 23 years diagnosis of AHC	Na n,K n, ↑ ACTH, ↓cortisol, ↑aldosterone, ↑renin	Mother as a carrier, brother affected	c.1268delA
Ahmad I, 2007 [18]	2.2 years		↓Na, ↑K, ↑ ACTH, ↓cortisol response to ACTH test, ↑renin	Maternal grandmother and mother as carriers, one maternal uncle affected, two maternal uncles died probably affected	T265R
Chung ST, 2015 [19]	2 weeks	4 months	↓Na, ↑K, ↑ ACTH, ↓cortisol, ↑renin, aldosterone undetectable	Mother as a carrier	c.1094 T > C
Kyriakakis N, 2017 case 1 [20]	19 years	42 years	↓Na, K n, ↓cortisol, ↓aldosterone		c.775 T > C
Kyriakakis N, 2017 case 2 [20]	30 years	37 years	↓Na, K n, ↓cortisol at baseline and after ACTH test, ↓aldosterone		c.836C > T
Li N, 2017 case1 [21]	8 years	18 years	↑ ACTH, ↓cortisol, ↓aldosterone, ↑renin	Cousin affected	L262P
Li N, 2017 case 2 [21]	At birth	10 years	↑ ACTH, ↓cortisol, aldosterone n, ↑renin	Cousin affected	L262P
Li N, 2017 case 3 [21]	8 years	23 years	↑ ACTH, ↓cortisol, aldosterone n, renin undetermined		C368F
Li N, 2017 case 4 [21]	At birth	27 years	↑ ACTH, Na n, K n, cortisol n, ↓aldosterone, renin n		637delC
Li N, 2017 case 5 [21]	At birth	6 months	↑ ACTH, ↓cortisol, ↓aldosterone, ↑renin		652_653delAC
Li N, 2017 case 6 [21]	10 years	20 years	↑ ACTH, ↓cortisol, ↓aldosterone, ↑renin		973delC
Li N, 2017 case 7 [21]	2 years	15 years	ACTH n, ↓cortisol, ↓aldosterone, renin n		774_775insCC
Li N, 2017 case 8 [21]	5 years	17 years	↑ ACTH, ↓cortisol, aldosterone and renin undetermined		L278P
Li N, 2017 case 9 [21]	11 years	26 years	↑ ACTH, ↓cortisol, ↓aldosterone, renin n		Q222X
Gerster K, 2017 case 1 [22]	2.5 years	2.5 years	↓Na, ↑K, ↑ ACTH, ↓cortisol, aldosterone and renin undetermined	Mother as a carrier	c.870C > A
Bizzarri C, 2016 case 1 [23]	21 days		↓Na, ↑K, ↑ ACTH, cortisol n, aldosterone n, ↑renin	Mother as a carrier, two sisters affected	P353LfsX387
Bizzarri C, 2016 case 2 [23]	21 days		↓Na, ↑K, ↑ ACTH, cortisol n, aldosterone n, ↑renin	Mother as a carrier, two sisters affected	P353LfsX387
Bizzarri C, 2016 case 3 [23]	3 days		↓Na, K n, ↑ ACTH, cortisol ↓, aldosterone n, ↑renin	Mother as a carrier, two sisters affected	P353LfsX387
Bizzarri C, 2016 case 4 [23]	8 days		↓Na, K ↑, ↑ ACTH, cortisol ↑, ↑renin	Mother as a carrier, two sisters affected	P353LfsX387
Al Amer AM, 2019 case 1 [24]	18 days	18 months	↓Na, K ↑, ACTH n, cortisol n, aldosterone n, ↑renin	Case 1 and 2 are identical twins	p.471 L > X
Al Amer AM, 2019 case 2 [24]	9.5 years	9.5 years	↓Na, K ↑, ACTH ↑, cortisol ↓, aldosterone and renin undetermined	Case 1 and 2 are identical twins	p.471 L > X

Replacement therapy with Hydrocortisone and Fludrocortisone guarantees an adequate growth of the *DAX-1* patients. The only case in which growth failure was detected despite optimization of therapy and nutrition was a patient with AHC and growth hormone deficiency (GHD) [19]. This underlines the importance of a genetic diagnosis as soon as possible, especially in this case where an isolated mineralocorticoid deficiency was misleading the diagnosis with a treatment by fludrocortisone only. The precocious diagnosis allowed to add hydrocortisone and will improve the future management of patient’s puberty, as hypogonadotropic hypogonadism may appear, and above all possible spermatogonia conservation before azoospermia development.

Although AHC onset is usually within the first 2 months of life, later and insidious manifestations of symptoms may occur in childhood. Ten cases of new adult-onset AHC have been described and diagnosis was suspected observing the association of adrenal insufficiency and hypogonadotropic hypogonadism [20].

The onset of puberty is variable in AHC but boys usually fail to enter puberty, gonadotropins levels are low, testosterone level can be normal or low and GnRH stimulation is usually ineffective. Some patients present transient manifestation of secondary sex precocity, due to gonadotropin-independent precocious puberty, ACTH-dependent precocious puberty, and gonadotropin-dependent central precocious puberty [25, 26]. However, usually exogenous gonadotropins fail in stimulating a complete pubertal development because of a primary defect in spermatogenesis. In AHC patients, testicular biopsy demonstrates hypoplasia of Sertoli cells and oligo-azoospermia [3, 19, 20]. *DAX-1* seems to increase the Gonadotropin-releasing hormone (GnRH) expression in the presence of SF-1 in a dose-dependent manner, consequently decreased expression of *DAX-1* could down-regulate GnRH secretion [27]. Thus, hormonal levels need to be monitored during the pubertal period and testosterone replacement therapy needs to be started, if puberty does not appear, to directly stimulate virilization.

Our patient shows levels of LH and FSH within the highest levels of normal range for 9 months of age, regular size of penis and testis, and mild high testosterone for age. We will follow up strictly his pubertal development, to avoid signs of precocious puberty, seen that the minipuberty profile seems delayed or longer than usual.

## Conclusion

To summarize, this case highlights that in newborn and infant with suspicion of congenital primary hypoaldosteronism, glucocorticoid function should be carefully assessed, possibly through a SST, with sometimes an increase of ACTH level preceding the cortisol defect but guiding to X-linked AHC. The phenotypical presentation may be variable and this underlines the importance of genetic diagnosis this leads to the correct monitoring of the patient (puberty, sterility) and his family by identifying the potential girls that are carriers.

## Data Availability

Not Applicable.

## Abbreviations

ACTH: Adrenocorticotropic hormone
AHC: Adrenal Hypoplasia Congenita
CAH: Congenital adrenal hyperplasia
ECG: Electrocardiogram
FSH: Follicle-stimulating hormone
GHD: Growth hormone deficiency
GnRH: Gonadotropin-releasing hormone
LH: Luteinizing hormone
MR: Mineralocorticoid receptor
MRI: Magnetic Resonance
SST: Short synacthen test
VLCFA: Very low chain fatty acids
